# Bioreactor as the root cause of the “manganese effect” during *Aspergillus niger* citric acid fermentations

**DOI:** 10.3389/fbioe.2022.935902

**Published:** 2022-08-04

**Authors:** Erzsébet Fekete, Vivien Bíró, Alexandra Márton, István Bakondi-Kovács, Zoltán Németh, Erzsébet Sándor, Béla Kovács, István Fábián, Christian P. Kubicek, Adrian Tsang, Levente Karaffa

**Affiliations:** ^1^ Department of Biochemical Engineering, Faculty of Science and Technology, University of Debrecen, Debrecen, Hungary; ^2^ Juhász-Nagy Pál Doctoral School of Biology and Environmental Sciences, University of Debrecen, Debrecen, Hungary; ^3^ Institute of Food Science, Faculty of Agricultural and Food Science and Environmental Management, University of Debrecen, Debrecen, Hungary; ^4^ Department of Inorganic and Analytical Chemistry, Faculty of Science and Technology, University of Debrecen, Debrecen, Hungary; ^5^ MTA-DE Redox and Homogeneous Catalytic Reaction Mechanism Research Group, Debrecen, Hungary; ^6^ Institute of Chemical, Environmental and Bioscience Engineering, TU Wien, Vienna, Austria; ^7^ Centre for Structural and Functional Genomics, Concordia University, Montreal, QC, Canada; ^8^ Institute of Metagenomics, University of Debrecen, Debrecen, Hungary

**Keywords:** *Aspergillus niger*, manganese ions, citric acid, stainless steel, metal ions leaching, fungal morphology

## Abstract

High-yield citric acid production by the filamentous Ascomycete fungus *Aspergillus niger* requires a combination of extreme nutritional conditions, of which maintaining a low manganese (II) ion concentration (<5 μg L^−1^) is a key feature. Technical-scale production of citric acid predominantly uses stainless-steel tank fermenters, but glass bioreactors used for strain improvement and manufacturing process development also contain stainless steel components, in which manganese is an essential alloying element. We show here that during citric acid fermentations manganese (II) ions were leaching from the bioreactor into the growth media, resulting in altered fungal physiology and morphology, and significant reduction of citric acid yields. The leaching of manganese (II) ions was dependent on the fermentation time, the acidity of the culture broth and the sterilization protocol applied. Manganese (II) ion leaching was partially mitigated by electrochemical polishing of stainless steel components of the bioreactor. High concentrations of manganese (II) ions during early cultivation led to a reduction in citric acid yield. However, the effect of manganese (II) ions on the reduction of citric acid yield diminished towards the second half of the fermentation. Since maintaining low concentrations of manganese (II) ions is costly, the results of this study can potentially be used to modify protocols to reduce the cost of citric acid production.

## 1 Introduction

Citric acid (2-hydroxy-propane-1,2,3-tricarboxylic acid) is a major commodity product of industrial biotechnology. It is extensively used in the food, chemical and pharmaceutical industries as pH-regulator, antioxidant, preservative, disinfectant and a flavoring agent. The bulk of the annual 2.3 million tons of citric acid production uses the filamentous Ascomycete fungus *Aspergillus niger* as the manufacturing host (for recent reviews, see Tong et al., 2019; Behera, 2020; Mores et al., 2021). Technical-scale citric acid production predominantly proceeds by submerged, aerobic fermentations in huge (>200 m^3^), agitated, stainless-steel tank reactors. High-yield citric acid production requires a combination of unusual culture conditions—high concentrations of a carbon source (>10%, w/v) with rapid uptake and catabolism, growth medium pH < 2, dissolved oxygen (DO) levels >30%, low concentrations of phosphate and several cations—which synergistically influence the yield (Shu and Johnson, 1948a; Röhr et al., 1996). These conditions result in a fungal morphology typified by short, swollen mycelia aggregated into small (<0.5 mm diameter) pellet-like clumps (Cox and Thomas, 1992). High molar yields (Y_product/substrate_> 80%) of citric acid occur only when cultures overwhelmingly comprise such morphological forms (Cox et al., 1998; Papagianni and Mattey, 2006). The deficiency of manganese (II) ions (Mn^2+^) in the growth medium is particularly critical: concentrations >5 μg L^−1^ (= parts per billion, ppb) reduces final citric acid yield by some 25% (Kisser et al., 1980). It is therefore crucial to establish at the onset of a citric acid fermentation a Mn^2+^-limited environment in the bioreactor. However, the cultivation environment should not be Mn^2+^-free as it is used as a co-factor by a wide range of enzymes (oxidoreductases, transferases, hydrolases, lyases, isomerases, ligases) required for fungal growth (Smith et al., 2017; Antsotegi-Uskola et al., 2020).

The above-mentioned threshold concentration of Mn^2+^ is in such low amounts that they may be present in the water used for fungal cultivation in industrial production (Karaffa et al., 2015). Furthermore, the surface of the carbon sources (typically D-glucose or sucrose) efficiently adsorbs divalent cations which are dissolved into the growth media during preparation. A variety of methods have been used to remove or counteract the excess Mn^2+^ from the culture broth, the most widespread being the cation exchange of growth media (Röhr et al., 1996). Manganese can also be removed by precipitation with ferrocyanide (hexacyanoferrate) or chelation by phytic acid (Yuichi and Marvin, 1961; Clark et al., 1965; Lönnerdal, 2002). Excess Mn^2+^ can be counteracted by increasing the copper (II) ion concentration in the growth media, likely through interference with cellular Mn^2+^ uptake and homeostasis (Hockertz et al., 1987; Netik et al., 1997). Efforts to manipulate Mn^2+^ transport in *A. niger* has recently led to the identification of a manganese transporter-encoding gene *dmtA*, the deletion of which resulted in Mn^2+^-insensitive production of citric acid (Fejes et al., 2020).

Stainless steel is a family of iron-based alloys containing >10.5% chromium which—in conjunction with low carbon content—imparts resistance to corrosion and heat. As well, stainless steel contains 8–18% nickel and up to 2% manganese (Shackelford and William, 2001). Corrosion resistance is the basic feature of stainless steel, but it can still corrode when exposed to acids, saline, grease, moisture, organic solvents such as acetonitrile and methanol or prolonged exposure to heat (Ko et al., 2021). Corrosion is typically an oxidation process driven by the tendency of refined metals to transfer into their chemically more stable state—e.g., iron converts to iron-oxide (rust)—and requires the simultaneous presence of moisture and oxygen. Since the chromium in the steel reacts with oxygen to form a thin, stable oxide layer on the surface that acts as a protective barrier, oxygen and water cannot access the underlying parts under mild environmental conditions. However, chemicals and high temperature can make exposed stainless steel lose their protective chromium-oxide layer. Since all the metal components of bioreactors used to produce citric acid are built of stainless steel, corrosion of the surface may lead to metal ion, including Mn^2+^, leaching in the course of the fermentation.

Electrochemical polishing can prevent or significantly reduce the leaching of metal ions. In this process, the corroded metal workpieces are immersed in a mixture of sulfuric and phosphoric acids, and connected as the positively-charged anode. A direct current passes from the anode to the negatively charged cathode where a reduction reaction produces hydrogen. Simultaneously, metals on the surface of the workpieces are oxidised and dissolved in the electrolyte, improving surface smoothness (Łyczkowska-Widłak et al., 2020). An alternative to electropolishing is the non-electrolytic method of steel passivation, which increases the corrosion resistance by removing free iron and foreign matter from the surface by nitric- or citric acid (Dai et al., 2021).

In this study, we quantified Mn^2+^ ion leaching from the stainless steel components of 6-L-scale glass bioreactors into the growth media during optimized *A. niger* citric acid fermentations, and identified heat sterilization and acidic culture pH as two of the underlying causes. Furthermore, we provide evidence to show that the adverse effect of Mn^2+^ on citric acid yield occurs during early stages of fermentation and diminishes as cultivation progresses.

## 2 Materials and methods

### 2.1 *Aspergillus niger* strains, media and cultivation conditions

*Aspergillus niger* NRRL2270 (A60; ATCC 11414), a hyper-producing strain frequently used for citric acid accumulation research (Perlman et al., 1946), was maintained at 4°C as conidiospores on agar plates containing minimal medium (pH 6): 10 g D-glucose L^−1^, 6 g NaNO_3_ L^−1^, 1.5 g KH_2_PO_4_ L^−1^, 0.5 g MgSO_4_*7 H_2_O L^−1^, and 0.5 g KCl L^−1^, supplemented with 20 µl trace element solution (containing, per litre: 10 g EDTA, 4.4 g ZnSO_4_ * 7 H_2_O, 1.01 g MnCl_2_ * 4 H_2_O, 0.32 g CoCl_2_ * 6 H_2_O, 0.315 g CuSO_4_ * 5 H_2_O, 0.22 g (NH_4_)_6_Mo_7_O_24_ * 4 H_2_O, 1.47 g CaCl_2_ * 7 H_2_O, 1.1 g FeSO_4_ * 7H_2_O).

Seed cultures were inoculated with 5 × 10^6^ *A. niger* conidia per mL of growth medium from a freshly prepared, high-density spore suspension in a 0.01% Tween 20 solution. Seed cultures were grown for 24 h in 500-ml Erlenmeyer (conical) flasks (VWR International Kft., Debrecen, Hungary) containing 100 ml of media in a rotary shaker (Infors AG, Basel, Switzerland) operating at 250 rpm at 30°C. Seed culture medium contained D-glucose as a sole carbon source at an initial level of 10 g L^−1^, and additionally contained 2.50 g (NH_4_)_2_SO_4_; 0.15 g KH_2_PO_4_; 0.15 g NaCl; 2.25 g MgSO_4_*7H_2_O; 1.50 mg Zn^2+^; 0.10 mg Fe^2+^ and 0.06 mg Cu^2+^ per litre. The initial medium pH was set at 3.0 with 3 M HCl, and was not controlled during the shake-flask cultivations.

Production cultures were grown in a chemically defined medium identical to the seed culture medium except that the initial D-glucose concentration was set at 140 g L^−1^ (Fejes et al., 2020). To control the concentration of Mn^2+^ in the growth medium, D-glucose was dissolved in distilled water and passed through a column (440 × 45 mm) of Dowex 50 W-X8 (100/200) cation exchange resin. All components were added to this d-glucose solution from sterile stock solutions. The final Mn^2+^ concentration was adjusted with MnCl_2_ * 4 H_2_O. The growth media thus prepared were membrane-filtered under aseptic conditions into the heat-sterilized and cooled shake-flasks or fermenters, containing the necessary volume of ion-exchanged and subsequently double-distilled water (henceforth referred to as Mn^2+^-free water). All chemicals used were analytical grade and purchased from Sigma-Aldrich Ltd (Budapest, Hungary).

Citric acid fermentations were carried out in batch mode either in 500 ml Erlenmeyer shake-flasks under conditions identical to those set up for the seed cultures (see above) or in two 6-L-scale autoclavable glass fermenters (Sartorius Biostat B, Göttingen, Germany, and Inel Ltd., Budapest, Hungary), henceforth referred to as Bioreactor A (Sartorius) and Bioreactor B (Inel). The two bioreactors share the same vessel geometry and working volume (4.5 L), their metal components are all made of AISI^1^ 316L (≤2% Mn^2+^ content)—the most commonly used grade of stainless steel in biotechnological applications (Zhou et al., 2011)—and both are equipped with a pair of six-blade Rushton-type disc turbine impellers of 65 mm diameter. However, Bioreactor A was newly purchased just before this study, while Bioreactor B was purchased some 10 years ago, and have been extensively used ever since, resulting with surface-type corrosion of the stainless steel parts (Supplementary Figure S1). During this study, all stainless steel components (headplate, sensor housings, agitator shaft, sampling tube, impellers) of Bioreactor B were subjected to electrochemical polishing (carried out by Zolend Ltd., Debrecen, Hungary) to examine its effect on leaching.

Standard bioreactor sterilization is defined here as autoclaving the vessels containing the necessary amount of Mn^2+^-free water up to an internal overpressure of 1.1 bar for a period of 30 min followed by the addition of ion-exchanged D-glucose and other sterilized medium components. In empty-vessel sterilization, the vessels contained a minimal amount of Mn^2+^-free water to cover the tips of the pH- and DO sensors. Following cooling, water was forced out by overpressure and the vessels were filled to the required levels with sterilized growth medium under aseptic conditions.

Both bioreactors were run using the same DO and temperature setpoints. Operating conditions were 30°C and 0.75 vessel volume per minute (vvm) of aeration. The initial pH of the growth medium was adjusted to 3.0 with 3M HCl before inoculation. The pH was measured and—depending on the experiment—was either controlled at set values as indicated, or was not controlled during fermentations. Dissolved oxygen levels were maintained at 30% saturation by adjusting the impeller(s) tip speed. Temperature, DO and impeller tip speed were controlled automatically by the controller units of the bioreactors. To minimize medium loss, the waste gas from the headspace was cooled in a reflux condenser connected to an external cooling bath (4°C) before exiting the system. Fermentations were inoculated under aseptic conditions with harvested and washed biomass from 500 ml of seed culture, and were run until the initial D-glucose of 140 g L^−1^ was depleted.

### 2.2 Analytical methods

Mycelial dry cell weight (DCW) was determined from 5 ml culture aliquots as described (Kozma and Karaffa, 1996). The biomass was harvested on a pre-weighed glass wool filter, washed with cold tap water, and dried at 80 °C until constant weight was obtained.

The concentrations of D-glucose and citric acid in the growth media were determined by high-pressure/performance liquid chromatography (HPLC; Agilent Technologies 1,260 Infinity II, United States) with a H^+^ exchange column (Bio-Rad Aminex HPX-87H^+^) at T = 55°C, using isocratic elution with 10 mM H_2_SO_4_ and refractive index detection (Karaffa et al., 1997; Fejes et al., 2020).

Mn^2+^ concentrations in the culture broth were determined by inductively coupled plasma quadrupole mass spectrometry (ICP-QMS; Thermo Fisher Scientific, Bremen, Germany) equipped with Hexapole Collision Cell Technology (Karaffa et al., 2015). Manganese (II) ion leaching rates were defined as the increase in Mn^2+^ concentration (ng L^−1^) per hour.

Biomass yield coefficients (Y_x/s_) were determined by dividing the maximal concentration of biomass (g L^−1^) achieved during fermentation by the initial carbon source (D-glucose) concentration (g L^−1^). Biomass production rates (g L^−1^ h^−1^) were calculated from the increase in DCW over the time elapsed between two consecutive samplings (i.e., sampling time points); the highest value obtained was taken to calculate the maximal specific growth rate of the culture (*μ*; h^−1^; (Pirt, 1975)). Likewise, D-glucose utilization rates (g L^−1^ h^−1^) were calculated from the steepest decrease in residual concentrations (g L^−1^) between two consecutive samplings. Specific molar citric acid yields (Y_p/s_) are the ratio between the moles of citric acid produced and the moles of D-glucose consumed after the complete depletion of the D-glucose. A high-yield citric acid fermentation (shake-flask or bioreactor alike) was defined as a culture reaching a Y_p/s_ of >0.8 (Karaffa et al., 2021).

Three aspects of mycelial morphology were monitored by microscopy: 1) swollen, yeast-like hyphae; 2) filamentous hyphae; and 3) pellet-like mycelial clumps (aggregates) (Bartoshevich et al., 1990; Paul and Thomasn, 1998; Sándor et al., 2021). To improve image contrast, lactophenol cotton blue (Fluka Chemie, Buch, Switzerland) at a final concentration of 10% (v/v) was added to the samples. Images were captured with a Zeiss Axio Imager phase-contrast microscope equipped with an AxioCam MRc 5 camera, and analysed with an AxioVision AC quantitative image analyser system. Average hyphal and pellet diameters (also referred to as micro- and macro-morphology, respectively) were assessed by processing >50 hyphae or >10 pellets per sample.

### 2.3 Reproducibility

All presented data involving fungal cultivations are the means of three independent experiments (biological replicates: starting with liquid cultures using different spore inocula), and each primary data is the mean of two parallel measurements within the same experiment (technical replicates). Data were analyzed and visualized with Sigmaplot software (Jandel Scientific, San Jose, CA, United States). The variability of the data was characterized by standard deviations for each procedure. Quantitative data (n ≥ 3) were compared using ANOVA (Analysis of Variance) with Holm-Sidak Test for pairwise comparisons. While probability (*p*) values were often <0.001, the criterion for significance was *p* < 0.05 in all cases.

## 3 Results

### 3.1 Characterization of the citric acid fermentations performed in bioreactors A and B

Following standard sterilization, we performed parallel citric acid fermentations in the two Bioreactors (A, B) under identical cultivation conditions. The sole carbon source was D-glucose, with initial concentrations set at 140 g L^−1^.

**FIGURE 1:**
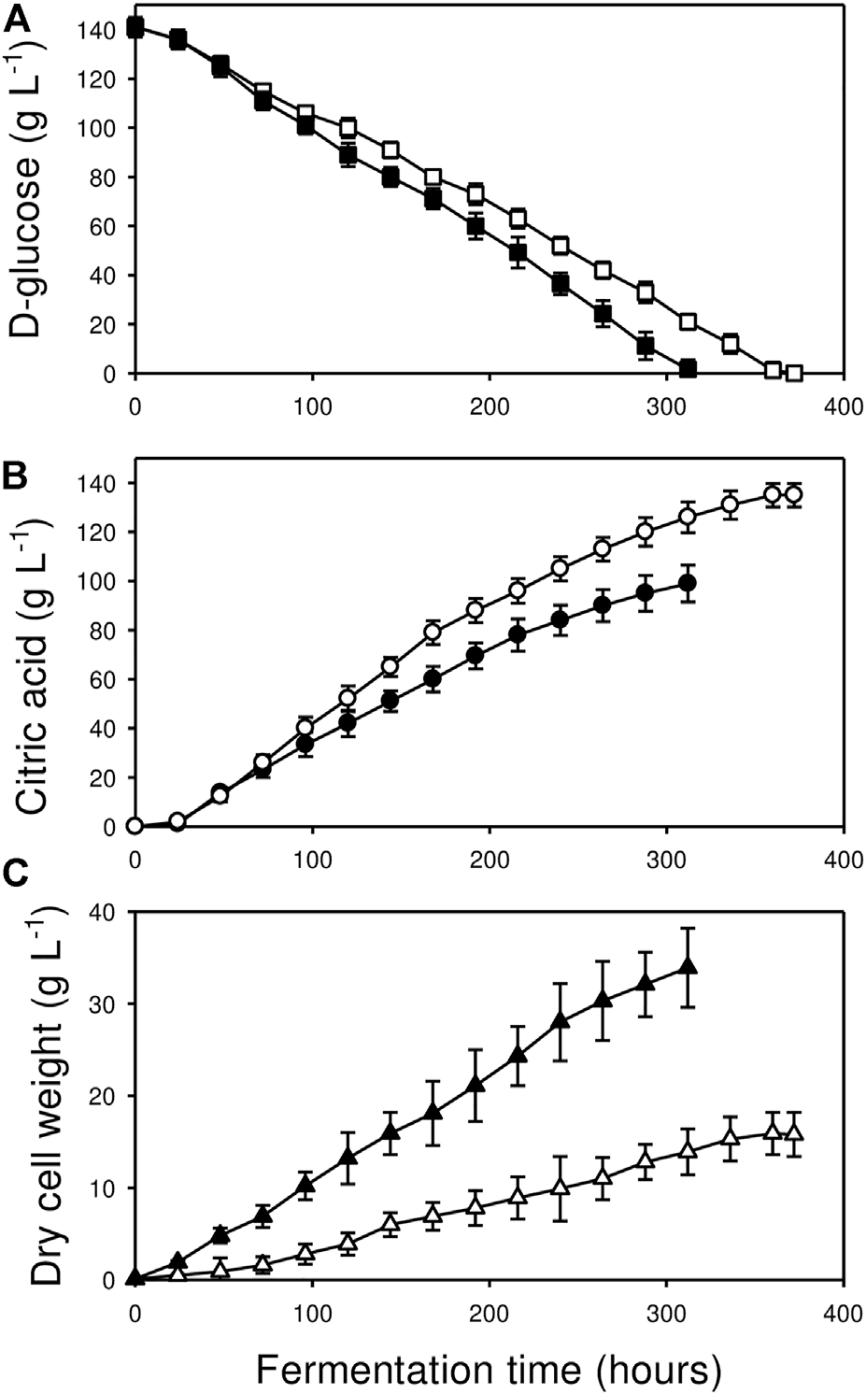
Kinetics of d-glucose utilization **(A)**; ■, □), citric acid production **(B)**; ●, O) and biomass formation **(C)**; ▲, △) in Bioreactor A (□, O, △) and in Bioreactor B (■, ●, ▲). Fermentations were carried out in triplicate. Standard deviations are indicated with vertical bars for each determined value. Note that the bar is sometimes smaller than the symbol that marks the mean concentration. See Materials and Methods for further details.

Despite of the identical starting conditions, the fermentations in the two bioreactors differed significantly in the four standard parameters (pH, biomass-, carbon substrate- and product concentrations) that were monitored (Figures 1, 3, Table 1). Residual D-glucose levels in Bioreactor B decreased more rapidly than in Bioreactor A, depleting the initial carbon substrate pool in 300 h as compared to 360 h. Final fungal biomass concentration in Bioreactor B was twice as high as Bioreactor A (33.9 vs. 15.9 g L^−1^), reflecting a higher overall specific growth rate for that culture (*μ* = 0.019 h^−1^ vs. 0.014 h^−1^). Particularly in the first 24 h, biomass in Bioreactor B grew significantly more rapidly, reaching a 19-fold higher concentration of the value measured right after inoculation, corresponding to a specific growth rate of *μ* = 0.12 h^−1^. In contrast, biomass concentration in Bioreactor A grew only five-fold higher over the same interval (*μ* = 0.067 h^−1^).

**TABLE 1:** D-glucose utilization rate, biomass (DCW) and citric acid (CA) production as well as derived kinetic parameters of *Aspergillus niger* NRRL 2270 cultivations in Bioreactors A and B (see Materials and Methods section for details). Mycelia grew under submerged conditions in an optimized CA-producing medium initially containing 140 g L^−1^ D-glucose as the sole carbon source.

	Maximal biomass concentration (g L^−1^)	Biomass yield (Y_x/s_)	Final CA concentration (g L^−1^)	Molar CA yield (Y_p/s_)	Maximal glucose utilization rate (g L^−1^ h^−1^)	Maximal biomass formation rate (g L^−1^ h^−1^)
Bioreactor A	15.9 ± 2.4	0.11 ± 0.01	135 ± 3.7	0.91 ± 0.02	0.50 ± 0.03	0.087 ± 0.01
Bioreactor B	33.9 ± 3.5	0.24 ± 0.01	99 ± 2.6	0.66 ± 0.04	0.58 ± 0.04	0.150 ± 0.03

Volumetric and molar yields of citric acid were, however, significantly higher in Bioreactor A than in B (Table 1). Our definition of high molar yield is Y_p/s_> 0.8 (Karaffa and Kubicek, 2003). Cultures grown in Bioreactor B were not considered high-yield fermentations (Y_p/s_ = 0.66 ± 0.04), while those grown in Bioreactor A produced outstanding molar citric acid yields (Y_p/s_ = 0.91 ± 0.02). As well, pH profiles were dissimilar, culminating in significant endpoint differences, pH 1.3 for the high-yield culture in Bioreactor A and pH 1.6 in Bioreactor B (Figure 3).

**FIGURE 2:**
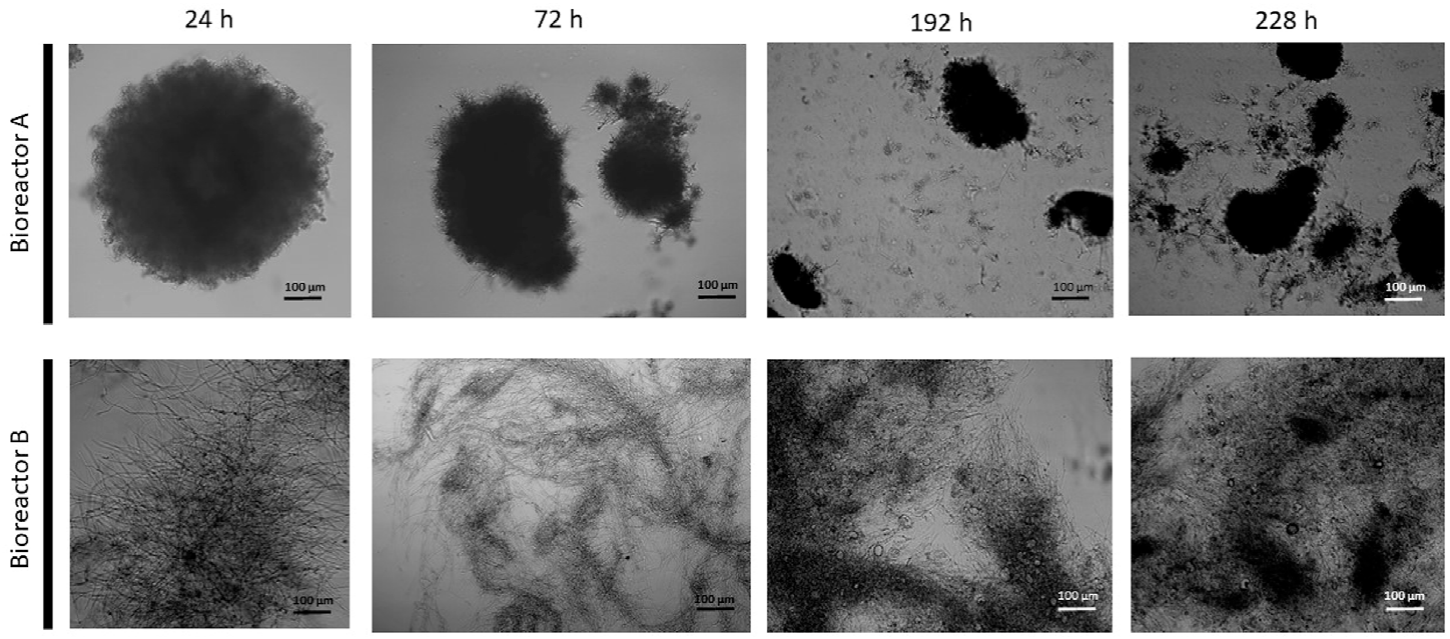
Microscopic images of submerged cultures of *Aspergillus niger* NRRL 2270. Images were taken at 24, 72, 192, and 228 h from each bioreactor.

Morphology of the fungal cultures in the two bioreactors also differed (Figure 2): the early stages (up until 168 h) in Bioreactor A were characterized by the dominance of small, pellet-like clumps (aggregates) and mycelia with short hyphae—typical for a high-yield citric acid producing cultures—as opposed to elongated hyphae in Bioreactor B, which is associated with low-yield citric acid producing cultures. In the later stages, cultures from the two bioreactors became more similar to each other with a generally pellet-like morphology (Figure 2). The high-producing forms have increased hyphal diameters (= micro-morphology) and reduced pellet diameters (= macro-morphology). These two quantitative parameters were assessed for variation during the fermentations (Table 2). In Bioreactor A, the average hyphal diameter in the 24 h old cultures was 5.98 ± 1.63 μm, which increased to 8.67 ± 1.66 μm at 360 h. In Bioreactor B, the respective maximal average hyphal diameters were significantly lower than in Bioreactor A, while diameters from early time-point samples were significantly smaller than the later ones. Average aggregate sizes significantly grew in the course of the fermentations in both bioreactors, and at each time-point they were significantly larger in Bioreactor B than in Bioreactor A.

**TABLE 2:** Average cell diameter and average aggregate size of *A. niger* NRRL2270 mycelia as a function of the sampling time (hours) in Bioreactor A and Bioreactor B during citric acid fermentations. All results are given in micrometer (μm).

	Bioreactor A		Bioreactor B	
Sampling time	Average cell diameter	Average aggregate size	Average cell diameter	Average aggregate size
**24**	5.98 ± 1.63	68 ± 11	1.92 ± 0.48	218 ± 81
**48**	6.56 ± 1.50	79 ± 15	1.90 ± 0.49	298 ± 96
**72**	7.12 ± 1.75	74 ± 13	2.10 ± 0.51	306 ± 78
**96**	7.01 ± 1.48	75 ± 20	2.28 ± 0.55	>350
**120**	6.78 ± 1.49	80 ± 12	2.05 ± 0.68	>350
**144**	8.12 ± 1.84	84 ± 10	2.90 ± 0.49	>350
**168**	7.76 ± 1.90	96 ± 13	2.48 ± 0.67	>350
**192**	7.99 ± 1.48	102 ± 16	2.87 ± 0.74	>350
**216**	7.12 ± 1.75	125 ± 21	3.23 ± 0.99	>350
**240**	8.05 ± 1.85	138 ± 24	3.90 ± 0.87	>350
**264**	8.56 ± 1.90	185 ± 21	3.90 ± 0.71	>350
**288**	8.88 ± 2.01	201 ± 28	4.14 ± 0.75	>350
**312**	8.76 ± 1.86	222 ± 32	4.25 ± 0.77	>350
**336**	8.99 ± 1.54	268 ± 48	4.68 ± 1.01	>350
**360**	8.67 ± 1.66	301 ± 68	5.02 ± 0.99	>350

In summary, cultures in the newly purchased Bioreactor A were excellent citric acid producers and displayed all the characteristic attributes of overflow metabolism, while those growing in the old Bioreactor B channelled a much higher part of the available carbon pool into biomass, significantly reducing molar citric acid yield.

### 3.2 Manganese ion concentrations in culture broths following sterilization and during fermentation

The difference in citric acid production yields and morphology between Bioreactors A and B led us to suspect that the concentrations of extracellular Mn^2+^ were different in the two bioreactors. The culture media were prepared in the same way with a final concentration of 2 μg L^−1^ Mn^2+^. Table 3 shows the Mn^2+^ concentrations for both bioreactors before and after heat sterilization. As expected, the concentrations of Mn^2+^ of the culture media before sterilization for both bioreactors were ∼2 μg L^−1^. Following 30 min of heat sterilization, the concentration of Mn^2+^ in Bioreactor A increased by ∼41% whereas that of Bioreactor B jumped by 10-fold. After 60 min of heat sterilization, the concentration of Mn^2+^ in Bioreactor A was 44% higher than before sterilization whereas in Bioreactor B the concentration was 13 times higher than before sterilization. These results suggest that heat sterilization caused a release of Mn^2+^ from the bioreactors and that the leaching was substantially more severe for the old bioreactor (B) than the new bioreactor (A).

**TABLE 3:** Manganese (II) ion concentrations (μg L^−1^) in Bioreactors A and B before and after 30 and 60 min of sterilizations in steam autoclave and the difference of the mean concentrations.

	Before sterilization	After sterilization		Differences of the means
		30 min	60 min	
Bioreactor A	2.05 ± 0.10	2.90 ± 0.5	2.95 ± 0.4	0.85/0.90
Bioreactor B	2.01 ± 0.18	20.30 ± 2.48	25.98 ± 3.02	18.29/23.97

**FIGURE 3:**
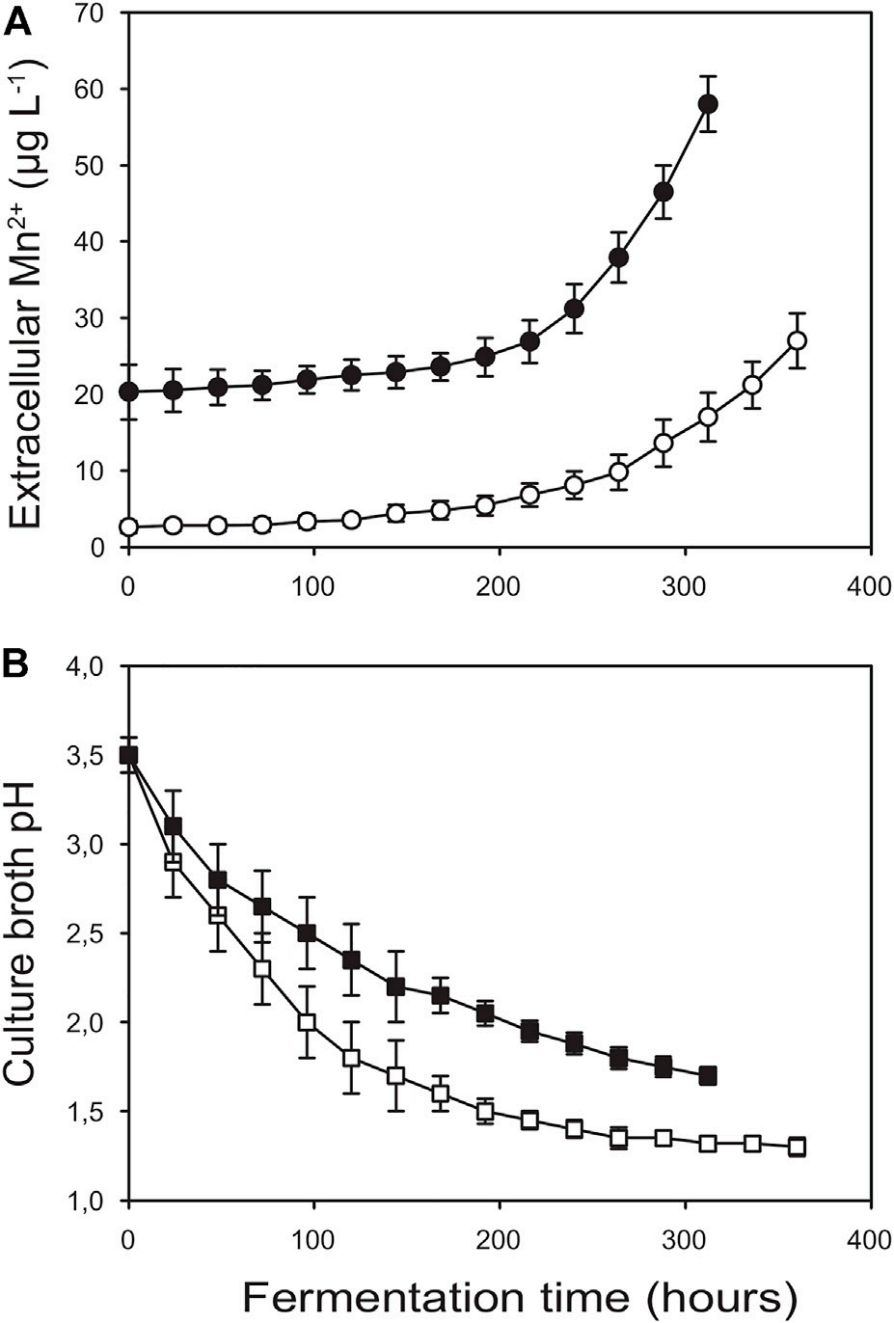
Kinetics of extracellular manganese(II) ion concentration **(A)** and extracellular pH **(B)** for Bioreactor A (□, O) and Bioreactor B (■, ●). The data were obtained for the fermentations shown in Figure 1.

Figure 3 shows the extracellular Mn^2+^ concentrations throughout the fermentations in Bioreactors A and B. In general, Mn^2+^ levels increased with time, and their kinetic profiles displayed a biphasic character. In the first phase that—at least in Bioreactor B—covered roughly the first half of the fermentations, leaching rates were lower and fairly constant. In the second phase, leaching rates steeply accelerated (Table 4). This pattern was less pronounced in Bioreactor A than in Bioreactor B. Importantly, Mn^2+^ concentrations in Bioreactor A remained below or around 5 μg L^−1^ for the entire first half of the fermentation, while in Bioreactor B—mostly due to the sharp increase after autoclaving the vessels—they were well above this threshold level throughout the fermentation.

**TABLE 4:** Mn^2+^ leaching rates (ng L^−1^ h^−1^) in Bioreactors A and B at culture broth pH > 2 and culture broth pH < 2 during citric acid fermentations by *A. niger* NRRL 2270.

	pH > 2	pH < 2	Entire fermentation
Bioreactor A	7.3 ± 0.6	90 ± 8.5	67.7 ± 6.2
Bioreactor B	23.9 ± 1.6	275.8 ± 18.9	120.8 ± 10.5

### 3.3 pH-dependence of manganese (II) ion leaching

**FIGURE 4:**
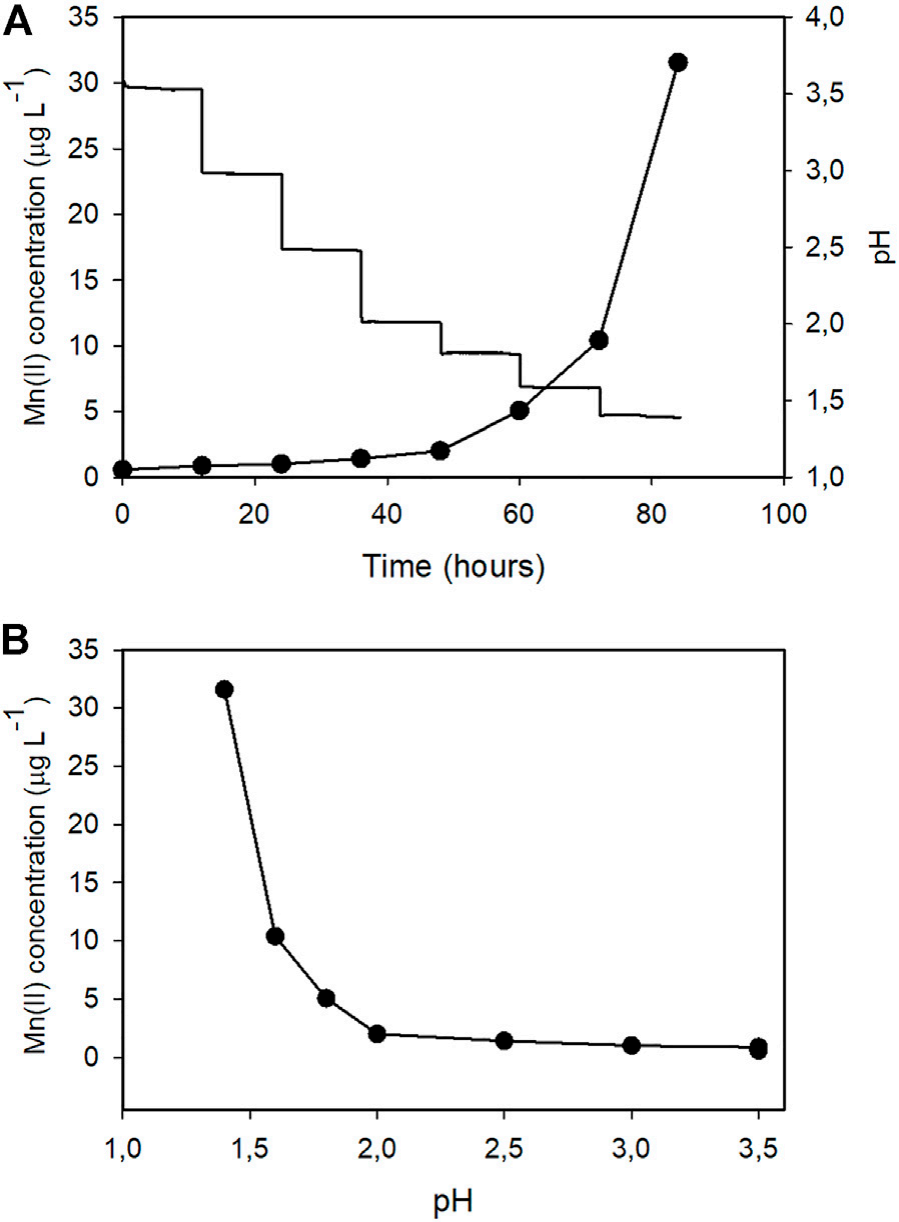
**A and B**Representations of the relationship between external pH and manganese (II) ion leaching in Bioreactor A. The growth medium was replaced by 10 mM phosphate-buffer solution. Temperature and dissolved oxygen levels were identical to those during the *A. niger* citric acid fermentations. The pH was adjusted by HCl at 12-h intervals from the initial pH 3.5 to pHs 3.0, 2.5, 2.0, 1.8, 1.6, and 1.4.

By comparing the kinetic profiles of the culture pH with that of the extracellular Mn^2+^ concentrations (Figure 3) we noticed that in both bioreactors Mn^2+^ accumulation significantly increased when the pH of the culture broth fell below ∼2.0. To investigate the relationship between pH and Mn^2+^ leaching, we filled Bioreactor A with either 10 mM or 100 mM phosphate-buffered Mn^2+^-free water. We ran the fermentation cycle using the same stirring speed and temperature as the fermentation cycle described in Figure 1, except that we controlled the pH with hydrochloric acid starting at pH 3.5 and dropping at 12-h intervals to pHs 3.0, 2.5, 2.0, 1.8, 1.6, and 1.4 (Figure 4). The results confirmed that the accumulation of Mn^2+^ is pH-dependent: negligible between 3.0 and 2.2, started to increase at 2.0, and steeply accelerated below pH 2.0, thereby showing a similarly two-phase nature as observed during the citric acid fermentations (Table 5). The Mn^2+^ leaching rates were similar to those measured during the fungal fermentations and were not statistically different at the two different concentrations of phosphate ions.

**TABLE 5:** Mn^2+^ leaching rates (ng L^−1^ h^−1^) at different pH levels in Bioreactor A. Measurements were performed in either 10 mM or 100 mM phosphate buffer solution prepared with double-destilled and ion-exchanged water. Temperature, dissolved oxygen levels and mechanical stirring rates were identical to those of a citric acid fermentation. pH was set by 1M hydrochloric acid.

pH	Mn^2+^ leaching rates	
	10 mM phosphate	100 mM phosphate
3.5	9.6 ± 0.3	10.4 ± 0.2
3.0	11.9 ± 1.3	12.6 ± 1.4
2.5	14.1 ± 2.2	15.2 ± 2.4
2.2	24.3 ± 2.1	26.9 ± 2.4
2.0	49.5 ± 2.5	54.3 ± 3.2
1.8	255.6 ± 12	238.7 ± 23
1.6	826.6 ± 48	845.7 ± 32
1.4	1766 ± 105	1687 ± 120

### 3.4 Prevention of manganese (II) ion leaching during citric acid fermentations

**FIGURE 5:**
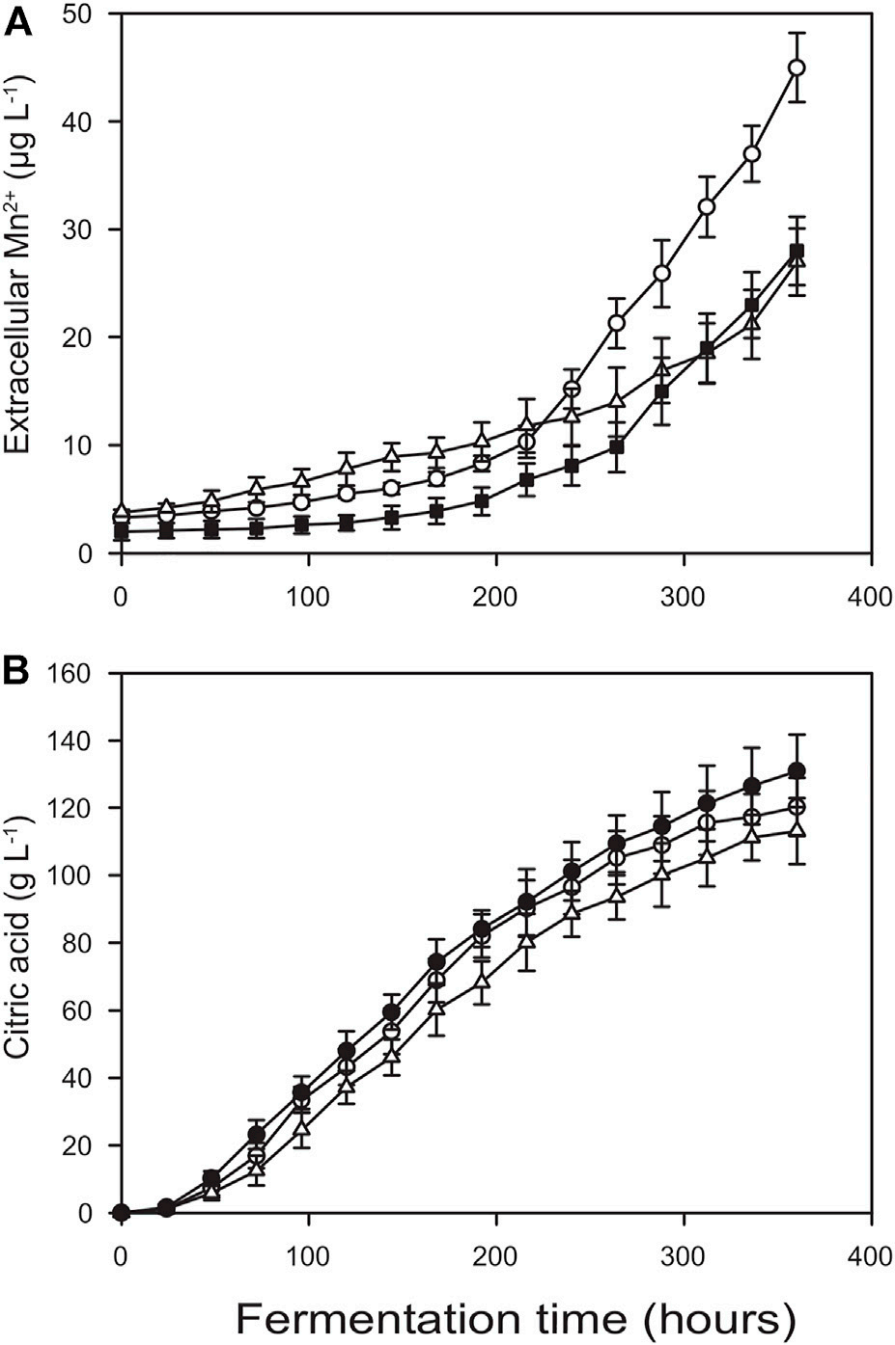
Kinetics of extracellular manganese (II) ion concentration **(A)** and citric acid concentration **(B)** in Bioreactor B after using empty vessel sterilization instead of standard autoclaving (O), after applying electrochemical polishing prior fermentation (△) and combining the two methods (■).

One of the means that was undertaken to mitigate Mn^2+^ leaching is an autoclaving method called empty vessel sterilization. In this process the minimal amount of water that harbours the leachable metal ions after the completion of the autoclave cycle is replaced with Mn^2+^-free sterile water. By this way, the early jump in the Mn^2+^ levels could be almost completely eradicated, although leaching rates after the inoculation remained similar (Figures 5 vs. Figure 3).

Another method to minimize metal ion leaching was the electrochemical polishing of the stainless steel components, as demonstrated for Bioreactor B. While the kinetic profiles of Mn^2+^ leaching still indicated considerable release both during autoclaving and the subsequent fermentation, the threshold value of 5 μg L^−1^ was exceeded only 48 h after inoculation. Consequently, citric acid molar yields were only ∼10% (though statistically still significantly) less than those attained in Bioreactor A, reaching a Y_p/s_ = 0.80 ± 0.03. Finally, combining these two latter methods restored citric acid molar yields to Y_p/s_ = 0.88 ± 0.04 even in (electrochemically polished) Bioreactor B (Figure 5). These value are statistically identical to those obtained in Bioreactor A.

### 3.5 The “manganese effect” on citric acid yield is dependent on the fermentation stage

Previous reports indicate that extracellular Mn^2+^ concentrations higher than 5 μg L^−1^ influence fungal physiology and morphology, and consequently citric acid yield. Figure 3 shows that the Mn^2+^ concentrations in Bioreactor A exceeded 5 μg L^−1^ after 168 h of fermentation and reached ∼30 μg L^−1^ by the end of the fermentations. The high Mn^2+^ concentrations at the later stages of fermentation did not reduce citric acid production (Figures 1, 3), suggesting that the adverse effects of Mn^2+^ on citric acid production occurs during the early stages of the fermentations. To test whether the adverse effect of Mn^2+^ on citric acid yield is indeed dependent on the cultivation stage, a set of shake-flask cultures were supplemented with 5, 30, and 100 μg L^−1^ Mn^2+^, respectively, at different time-points (Table 6). 30 μg L^−1^ Mn^2+^ is the concentration in Bioreactor A towards the end of the fermentations, while 100 μg L^−1^ already saturates the Mn^2+^ uptake system of *A. niger* NRRL2270 (Fejes et al., 2020). Results confirmed that exposure to higher concentrations of Mn^2+^ during early cultivation resulted in the reduction of citric acid yields and the simultaneous increase of fungal biomass. Both effects were dependent on the concentration of the supplemented Mn^2+^, and both gradually diminished towards the second half of the fermentation.

**TABLE 6:** Effect on manganese (II) ion supplementation on citric acid molar yield (Y_p/s_) and biomass formation yield (Y_x/s_) as a function of time during citric acid fermentation with *A. niger* NRRL2270. Control cultures contained an initial concentration of 2 μg L^−1^ manganese (II) ions. Product and biomass yields for the control cultures: Y_p/s_ (%) = 90.1 ± 2.5 and Y_x/s_ (%) = 18.5 ± 1.8.

Manganese (II) ion supplementation (h)	5 μg L^−1^		30 μg L^−1^		100 μg L^−1^	
	Y_p/s_	Y_x/s_	Y_p/s_	Y_x/s_	Y_p/s_	Y_x/s_
**0**	0.73 ± 0.05	0.25 ± 0.04	0.66 ± 0.04	0.25 ± 0.05	0.35 ± 0.02	0.36 ± 0.04
	*p* < 0.05	*p* > 0.05	*p* < 0.05	*p* > 0.05	*p* < 0.05	*p* < 0.05
**3**	0.67 ± 0.05	0.27 ± 0.04	0.62 ± 0.05	0.26 ± 0.05	0.33 ± 0.03	0.35 ± 0.04
	*p* < 0.05	*p* > 0.05	*p* < 0.05	*p* > 0.05	*p* < 0.05	*p* < 0.05
**6**	0.71 ± 0.04	0.26 ± 0.05	0.63 ± 0.03	0.25 ± 0.03	0.39 ± 0.04	0.38 ± 0.05
	*p* < 0.05	*p* > 0.05	*p* < 0.05	*p* > 0.05	*p* < 0.05	*p* < 0.05
**12**	0.70 ± 0.03	0.27 ± 0.04	0.64 ± 0.04	0.26 ± 0.04	0.40 ± 0.05	0.33 ± 0.05
	*p* < 0.05	*p* > 0.05	*p* < 0.05	*p* > 0.05	*p* < 0.05	*p* < 0.05
**24**	0.72 ± 0.06	0.25 ± 0.04	0.64 ± 0.03	0.24 ± 0.05	0.44 ± 0.04	0.35 ± 0.04
	*p* < 0.05	*p* > 0.05	*p* < 0.05	*p* > 0.05	*p* < 0.05	*p* < 0.05
**48**	0.78 ± 0.04	0.23 ± 0.04	0.65 ± 0.05	0.25 ± 0.05	0.52 ± 0.05	0.30 ± 0.04
	*p* < 0.05	*p* > 0.05	*p* < 0.05	*p* > 0.05	*p* < 0.05	*p* < 0.05
**72**	0.83 ± 0.05	0.16 ± 0.03	0.77 ± 0.02	0.20 ± 0.03	0.71 ± 0.04	0.21 ± 0.04
	*p* > 0.05	*p* > 0.05	*p* < 0.05	*p* > 0.05	*p* < 0.05	*p* > 0.05
**172**	0.88 ± 0.04	0.12 ± 0.03	0.89 ± 0.02	0.13 ± 0.03	0.87 ± 0.04	0.16 ± 0.03
	*p* > 0.05	*p* > 0.05	*p* > 0.05	*p* > 0.05	*p* > 0.05	*p* > 0.05

## 4 Discussion

Mn^2+^ concentrations of <5 μg L^−1^ in the *A. niger* culture medium is a key condition for achieving a high-yield during citric acid fermentation (Kisser et al., 1980). The task is challenging since even tap water may contain up to 3 μg L^−1^ Mn^2+^, a 1% (w/v) D-glucose solution contains the double concentration of the threshold, while the Mn^2+^ levels in a 15% (w/v) D-glucose solution exceeds it by 30-fold (Karaffa et al., 2015). In this paper we tested and confirmed the hypothesis that in addition to contamination in the growth medium, the bioreactor itself can also be the source of Mn^2+^ to such an extent that the corrosion-driven leaching could surpass the critical levels, reducing molar citric acid yield by ∼30%. We provided evidence that leaching of Mn^2+^ from the alloy of the stainless steel components is predominantly associated with standard autoclaving and the strongly acidic (pH < 2) culture broth characteristic for this fermentation technology. In addition, we also demonstrated that the application of anti-corrosion treatments and/or using a modified sterilization protocol fully restored high citric acid yield.

Although single-use bioreactors have gained acceptance in biotechnology for over two decades, their application is still limited to mammalian, insect and plant cell cultivations (Langer and Rader, 2018). Microbial bioprocesses have not been converted to disposables made of plastics/elastomers because of scale limitations mainly due to the relatively low oxygen transfer rates (Rader, 2014). Stainless steel bioreactors on the other hand can be customized up to several hundred cubic meters and still possessing higher mixing capacity than what single-use bioreactors are able to offer. Indeed, an estimated 97% of the world’s bioprocessing capacity is made of stainless steel (Langer and Rader, 2018). Glass bioreactors used for strain improvement and manufacturing process development contain several stainless steel components (agitation-aeration system, sampling tube, sensor/electrode housings, and baffles). Corrosion-driven metal ion leaching must therefore be a widespread phenomenon that nonetheless appears rarely considered for bioprocesses, at least in the public domain. Manganese leaching was reported to influence terminal galactosylation in monoclonal antibodies produced by CHO-K1SV cells, grown in 2-L scale glass bioreactors (Williamson et al., 2018).

The amount of metal ions leaching into the environment was described to be subject to multiple variables such as external pH, temperature, contact materials, exposed surface area and contact duration/cultivation time (Zhou et al., 2011; Williamson et al., 2018). Due to the experimental setup employed in this study, most variables (contact materials, contact area, cultivation time, temperature) can be considered as constants. The only meaningful difference between the newly purchased Bioreactor A and the old, used Bioreactor B was the advanced state of surface corrosion of the latter that, however, we did not attempt to characterize—either qualitatively or quantitatively—beyond what could be seen by naked eye (Supplementary Figure 1). Ultimately, the corrosion caused by many years of exposure to harsh physical conditions rendered Bioreactor B unsuitable for high-yield citric acid fermentations, as standard autoclaving (T = 121°C, t = 30 min) already raised Mn^2+^ levels above the threshold. However, electrochemical polishing, empty vessel sterilization, and the two methods together restored usability of Bioreactor B for high-yield citric acid production. These results clearly identify leaching from corroded stainless steel components as the root cause of the “manganese effect”. Likewise, zinc, chromium, and manganese leaching were all reported to be exacerbated in 2-L scale bioreactors when the glass vessel complete with all stainless steel components was autoclaved, while only zinc was detected after the vessel was autoclaved with a headplate only (Williamson et al., 2018).

Another factor that contributes to Mn^2+^ leaching is the low pH of the contact liquid. The release of metal ions from 316L stainless steel in dilute hydrochloric acid serves evidence for the degradation of these alloys (Atapour et al., 2020). Mn^2+^ leaching has not been investigated before, however, acidic conditions and possible complex formation reactions between Mn^2+^ and citric acid may enhance the dissolution process (Bastug et al., 2007; Wyrzykowski and Chmurzyński, 2010). A similar interpretation was proposed for the deteriorating effect of *Pseudomonas aeruginosa* on alpha-brass and 316L stainless steel (Farooq et al., 2021). However, the low pH (pH ≤ 2) during citric acid fermentation is critical to avoid production of unwanted byproducts such as oxalic acid (Ruijter et al., 1999). Externally controlling the culture pH at or above 2 is therefore not a viable option.

Mn^2+^ concentrations started to rise sharply when the culture broth pH dropped below about pH 2.0., and continued to increase as pH dropped to 1.4 (this value was never achieved in Bioreactor B, underlining the importance of very low pH in high-yield citric acid accumulation; (Kubicek et al., 1988). This relationship was qualitatively and quantitatively essentially identical in the culture broth and the phosphate buffer solution, indicating that the *A. niger* strain NRRL 2270 did not contribute to Mn^2+^ leaching. Proteins, complexing agents such as EDTA and buffer agents such as phosphate can nonetheless accelerate metal leaching from stainless steel (Allain and Wang, 2007). We therefore cannot exclude the possibility that phosphate ions did play some part in Mn^2+^ leaching. Yet the extent of this contribution was likely minor, as increasing phosphate concentration from 10 to 100 mM did not increase the Mn^2+^ leaching rate.

We did not monitor the leaching of other metals than Mn^2+^ in this study, hence the question arises whether other metal ions that are present in the 316L stainless steel can—at least partially—be responsible for the altered fungal metabolism and reduced citric acid production. While evidence-based answer cannot be given, several factors argue against this possibility. Iron has been discussed as an element reducing citric acid yield (Shu and Johnson, 1947; Shu and Johnson, 1948a). However, while iron—the most abundant metal in stainless steel—was supplemented here to the growth medium in 0.1 mg L^−1^ concentration, other citric acid production formulas advocate using up to 1.3 mg L^−1^ Fe^2+^ ions (Shu and Johnson, 1948b). Secondly, it is plausible—though not yet confirmed experimentally—that the inhibitory effect of iron in concentrations higher than above on the citric acid yield is actually related to the manganese effect, as even analytical grade iron salts may contain up to 0.5% (w/w) Mn^2+^ as impurities (Karaffa et al., 2021). Such Fe/Mn interactions obviously do not exist in our experimental setup. Thirdly, chromium—the second most abundant metal in 316L-grade stainless steel—was described to even stimulate citric acid production in concentrations up to 50 mg L^−1^ (Angumeenal et al., 2002; Angumeenal et al., 2003). While this favourable effect of chromium ions may be strain-specific, no reports to the contrary are available in the literature. Finally, Mn^2+^ deficiency transforms the filamentous hyphal morphology of *A. niger* to one dominated by “yeast-like cells” (branches of short, swollen forms) on the micro-morphology-, and small (<0.5 mm diameter) compact pellets on the macro-morphology level (Detroy and Ciegler, 1971; Gyamerah, 1995). Progression of this transformation is always accompanied by increased cell (hyphae) diameter and reduced pellet size (Paul et al., 1994; El-Sabbagh et al., 2008). These phenotypes are exactly what we have observed upon utilizing the corroded (B) and the non-corroded (A) bioreactors.

We demonstrated that under high-yield citric acid producing conditions even a brand-new bioreactor releases more Mn^2+^ into the culture broth than the treshold level, but this leaching does not limit citric acid accumulation as long as it occurs in the late stages of the fermentation. A more detailed investigation revealed that—at least under the conditions used in the experiments—manganese deficiency in the first 48 h of the cultivation appear critical for citric acid overflow. This time-span may turn out to be strain- and technology dependent, but the results indicate that the “manganese effect” diminishes as fermentation progresses, mitigating the problem of Mn^2+^ leaching from metal surfaces. The results also imply that once *A. niger* citric acid overflow commences due to the special cultivation conditions, it continues irrespective of the changing environment in the bioreactor. Indeed, the high initial concentration of d-glucose—considered one of the most critical elements of high-yield citric acid production—also gradually decreases as the fermentation progresses, but that does not seem to influence the sugar/acid conversion rate either. We thus hypothetize that the genes involved in the response to Mn^2+^ deficiency are expressed at a very early stage of the cultivation. We are currently testing this hypothesis.

## 5 Conclusion

During citric acid fermentations by *A. niger*, the stainless steel components of the bioreactor can be the source of manganese (II) ions to such an extent that the corrosion-driven leaching could surpass the critical levels, resulting in altered fungal physiology and morphology, and reduction of product yields by ∼30%. The leaching of manganese is dependent on the fermentation time, the acidity of the culture broth and the sterilization protocol applied. The effect of manganese (II) ions on the reduction of citric acid yield diminishes towards the second half of the fermentation. Since maintaining low concentrations of manganese (II) ions in the culture broth is costly, these results can potentially be used to modify protocols to reduce the cost of citric acid production.

## Data Availability

The original contributions presented in the study are included in the article/Supplementary Material, further inquiries can be directed to the corresponding author.

## Footnotes

1 American Iron and Steel Institute.

## Supplementary material

Supplementary Figure S1 Corroded stainless steel parts of Bioreactor B.

## References

[B1] Allain L. Wang Q. (2007). Impact of package leachables on the stability of pharmaceutical products. Am. Pharm. Rev. 10, 38–44.

[B2] Angumeenal A. Kamalakannan P. Prabhu H. J. Venkappayya D. (2003). Effect of transition metal cations on the production of citric acid using mixed cultures of *Aspergillus niger* and *Candida gulliermondii* . J. Indian Chem. Soc. 80, 903–906. 10.5281/zenodo.5839322

[B3] Angumeenal A. R. Kamalakannan P. Prabhu H. J. Yenkappayya D. (2002). Effect of transition metal cations and anions on the production of citric acid by *Aspergillus niger* . Indian J. Chem. Technol. 9, 508–512.

[B4] Antsotegi-Uskola M. Markina-Iñarrairaegui A. Ugalde U. (2020). New insights into copper homeostasis in filamentous fungi. Int. Microbiol. 23, 65–73. 10.1007/s10123-019-00081-5 31093811 PMC6981102

[B5] Atapour M. Wang X. Y. Farnlund K. Wallinder I. O. Hedberg Y. (2020). Corrosion and metal release investigations of selective laser melted 316L stainless steel in a synthetic physiological fluid containing proteins and in diluted hydrochloric acid. Electrochimica Acta 354, 136748. 10.1016/j.electacta.2020.136748

[B6] Bartoshevich Y. E. Zaslavskaya P. Novak M. Yudina O. (1990). *Acremonium chrysogenum* differentiation and biosynthesis of cephalosporin. J. Basic Microbiol. 30, 313–320. 10.1002/jobm.3620300503 2213533

[B7] Bastug A. S. Gokturk S. Sismanoglu T. (2007). 1: 1 binary complexes of citric acid with some metal ions: stability and thermodynamic parameters. Rev. Inorg. Chem. 27 (1), 53–65. 10.1515/REVIC.2007.27.1.53

[B8] Behera B. C. (2020). Citric acid from *Aspergillus niger*: a comprehensive overview. Crit. Rev. Microbiol. 46, 727–749. 10.1080/1040841X.2020.1828815 33044884

[B9] Clark D. S. Ito K. Tymchuk P. (1965). Effect of potassium ferrocyanide on the chemical composition of molasses mash used in the citric acid fermentation. Biotechnol. Bioeng. 7, 269–278. 10.1002/BIT.260070206

[B10] Cox P. W. Paul G. C. Thomas C. R. (1998). Image analysis of the morphology of filamentous microorganisms. Microbiology 144, 817–827. 10.1099/00221287-144-4-817 9579057

[B11] Cox P. W. Thomas C. R. (1992). Classification and measurement of fungal pellets by automated image analysis. Biotechnol. Bioeng. 39, 945–952. 10.1002/bit.260390909 18601032

[B12] Dai H. Shi S. Yang L. Guo C. Chen X. (2021). Recent progress on the corrosion behavior of metallic materials in HF solution. Corros. Rev. 39 (4), 313–337. 10.1515/corrrev-2020-0101

[B13] Detroy R. W. Ciegler A. (1971). Induction of yeastlike development in *Aspergillus parasiticus* . J. Gen. Microbiol. 65, 259–264. 10.1099/00221287-65-3-259 5556679

[B14] El-Sabbagh N. Harvey L. McNeil B. (2008). Effects of dissolved carbon dioxide on growth, nutrient consumption, cephalosporin C synthesis and morphology of *Acremonium chrysogenum* in batch cultures. Enzyme Microb. Technol. 42, 315–324. 10.1016/j.enzmictec.2007.10.012

[B15] Farooq A. Zubair M. Wadood H. Z. Deen K. M. (2021). Effect of *Pseudomonas aeruginosa* strain ZK biofilm on the mechanical and corrosion behavior of 316L stainless steel and alpha-brass. J. Electrochem. Sci. Technol. 12 (4), 431–439. 10.33961/jecst.2020.01718

[B16] Fejes B. Ouedraogo J. P. Fekete E. Sándor E. Flipphi M. Soós Á. (2020). The effects of external Mn^2+^ concentration on hyphal morphology and citric acid production are mediated primarily by the NRAMP-family transporter DmtA in *Aspergillus niger* . Microb. Cell Fact. 19, 17. 10.1186/s12934-020-1286-7 32000778 PMC6993379

[B17] Gyamerah M. (1995). Factors affecting the growth form of *Aspergillus terreus* NRRL 1960 in relation to itaconic acid fermentation. Appl. Microbiol. Biotechnol. 44, 356–361. 10.1007/BF00169929

[B18] Hockertz S. Schmid J. Auling G. (1987). A specific transport system for manganese in the filamentous fungus *Aspergillus niger* . Microbiology 133, 3513–3519. 10.1099/00221287-133-12-3513

[B19] Karaffa L. Díaz R. Papp B. Fekete E. Sándor E. Kubicek C. P. (2015). A deficiency of manganese ions in the presence of high sugar concentrations is the critical parameter for achieving high yields of itaconic acid by *Aspergillus terreus* . Appl. Microbiol. Biotechnol. 99, 7937–7944. 10.1007/s00253-015-6735-6 26078111

[B20] Karaffa L. Fekete E. Kubicek C. P. (2021). The role of metal ions in fungal organic acid accumulation. Microorganisms 9, 1267. 10.3390/microorganisms9061267 34200938 PMC8230503

[B21] Karaffa L. Kubicek C. P. (2003). *Aspergillus niger* citric acid accumulation: do we understand this well-working black box? Appl. Microbiol. Biotechnol. 61, 189–196. 10.1007/s00253-002-1201-7 12698275

[B22] Karaffa L. Sándor E. Kozma J. Szentirmai A. (1997). Methionine enhances sugar consumption, fragmentation, vacuolation and cephalosporin C production in *Acremonium chrysogenum* . Process Biochem. 32, 495–499. 10.1016/S0032-9592(97)00003-4

[B23] Kisser M. Kubicek C. P. Röhr M. (1980). Influence of manganese on morphology and cell wall composition of *Aspergillus niger* during citric acid fermentation. Arch. Microbiol. 128, 26–33. 10.1007/BF00422301 7458536

[B24] Ko G. Kim W. Kwon K. Lee T-K. (2021). The corrosion of stainless steel made by additive manufacturing: a review. Met. (Basel). 11 (3), 516. 10.3390/met11030516

[B25] Kozma J. Karaffa L. (1996). Effect of oxygen on the respiratory system and cephalosporin C production in *Acremonium chrysogenum* . J. Biotechnol. 48, 59–66. 10.1016/0168-1656(96)01400-9 8818273

[B26] Kubicek C. P. Schreferl-Kunar G. Wöhrer W. Röhr M. (1988). Evidence for a cytoplasmic pathway of oxalate biosynthesis in *Aspergillus niger* . Appl. Environ. Microbiol. 54, 633–637. 10.1128/aem.54.3.633-637.1988 3132096 PMC202517

[B27] Langer E. S. Rader R. A. (2018). Biopharmaceutical manufacturing is shifting to single-use systems. Are the dinosaurs, the large stainless steel facilities, becoming extinct? American Pharmaceutical Review. Art. No. 354820.

[B28] Lönnerdal B. (2002). Phytic acid-trace element (Zn, Cu, Mn) interactions. Int. J. Food Sci. Technol. 37, 749–758. 10.1046/j.1365-2621.2002.00640.x

[B29] Łyczkowska-Widłak E. Lochyński P. Nawrat G. (2020). Electrochemical polishing of austenitic stainless steels. Materials 13 (11), 2557. 10.3390/ma13112557 PMC7321480 32512733

[B30] Mores S. Vandenberghe L. P. D. S. Magalhães Júnior A. I. de Carvalho J. C. de Mello A. F. M. Pandey A. (2021). Citric acid bioproduction and downstream processing: status, opportunities, and challenges. Bioresour. Technol. 320, 124426. 10.1016/j.biortech.2020.124426 33249260

[B31] Netik A. Torres N. V. Riol J-M. Kubicek C. P. (1997). Uptake and export of citric acid by *Aspergillus niger* is reciprocally regulated by manganese ions. Biochim. Biophys. Acta Biomembr. 1326, 287–294. 10.1016/s0005-2736(97)00032-1 9218559

[B32] Papagianni M. Mattey M. (2006). Morphological development of *Aspergillus niger* in submerged citric acid fermentation as a function of the spore inoculum level. Application of neural network and cluster analysis for characterization of mycelial morphology. Microb. Cell Fact. 5 (3), 3. 10.1186/1475-2859-5-3 16433930 PMC1386700

[B33] Paul G. C. Thomas C. R. (1998). Characterisation of mycelial morphology using image analysis. Adv. Biochem. Eng. Biotechnol. 60, 1–59. 10.1007/BFb0102278 9468800

[B34] Paul G. Kent C. Thomas C. R. (1994). Hyphal vacuolation and fragmentation in *Penicillium chrysogenum* . Biotechnol. Bioeng. 44, 655–660. 10.1002/bit.260440513 18618802

[B35] Perlman D. Kita D. A. Peterson W. A. (1946). Production of citric acid from cane molasses. Arch. Biochem. 11, 123–129. 20998032

[B36] Pirt S. J. (1975). Principles of microbe and cell cultivation. Oxford, United Kingdom: Blackwell Scientific Publications, 156–170.

[B37] Rader R. A. (2014). Biopharmaceutical manufacturing: Historical and future trends in titers, yields, and efficiency in commercial-scale bioprocessing. BioProcess. J. 13 (4), 47–54. 10.12665/j134.langer

[B38] Röhr M. Kubicek C. P. Kominek J. (1996). “Citric acid,”in Biotechnology: products of primary metabolism. Editors Rehm H. J. Reed G. (Weinheim: Verlag Chemie), 6, 308–345.

[B39] Ruijter G. J. G. Vondervoortvan de P. J. Visser J. (1999). Oxalic acid production by *Aspergillus niger*: an oxalate-non-producing mutant produces citric acid at pH 5 and in the presence of manganese. Microbiology 145, 2569–2576. 10.1099/00221287-145-9-2569 10517610

[B40] Sándor E. Kolláth I. S. Fekete E. Bíró V. Flipphi M. Kovács B. (2021). Carbon-source dependent interplay of copper and manganese ions modulates the morphology and itaconic acid production in *Aspergillus terreus* . Front. Microbiol. 12, 680420. 10.3389/fmicb.2021.680420 34093503 PMC8173074

[B41] Shackelford J. F. William A. (2001). The CRC materials science and engineering handbook. 3rd ed. Boca Raton: CRC Press LLC.

[B42] Shu P. Johnson M. J. (1948b). Citric acid production by submerged fermentation with *Aspergillus niger* . Ind. Eng. Chem. 40, 1202–1205. 10.1021/ie50463a008

[B43] Shu P. Johnson M. J. (1947). Effect of the composition of the sporulation medium on citric acid production by *Aspergillus niger* in submerged culture. J. Bacteriol. 54, 161–167. 10.1128/jb.54.2.161-167.1947 PMC526531 16561343

[B44] Shu P. Johnson M. J. (1948a). The interdependence of medium constituents in citric acid production by submerged fermentation. J. Bacteriol. 56, 577–585. 10.1128/jb.56.5.577-585.1948 16561608 PMC518625

[B45] Smith A. D. Logeman B. L. Thiele D. J. (2017). Copper acquisition and utilization in fungi. Annu. Rev. Microbiol. 71, 597–623. 10.1146/annurev-micro-030117-020444 28886682 PMC6827982

[B50] Tong Z. Zheng X. Tong Y. Shi Y.-C. Sun J. (2019). Systems metabolic engineering for citric acid production by *Aspergillus niger* in the post-genomic era. Microb. Cell Fact. 18, 28. 10.1186/s12934-019-1064-6 30717739 PMC6362574

[B46] Williamson J. Miller J. McLaughlin J. Combs R. Chu C. (2018). Scale-dependent manganese leaching from stainless steel impacts terminal galactosylation in monoclonal antibodies. Biotechnol. Prog. 34, 1290–1297. 10.1002/btpr.2662 29885096

[B47] Wyrzykowski D. Chmurzyński L. (2010). Thermodynamics of citrate complexation with Mn^2+^, Co^2+^, Ni^2+^ and Zn^2+^ ions. J. Therm. Anal. Calorim. 102 (1), 61–64. 10.1007/s10973-009-0523-410.1007/s10973-009-0523-4

[B48] Yuichi N. Marvin J. J. (1961). Citric acid fermentation of sugars purified with chelating resin. J. Bacteriol. 82, 538–541. 10.1128/jb.82.4.538-541.1961 14480219 PMC279203

[B49] Zhou S. Schöneich C. Singh S. K. (2011). Biologics formulation factors affecting metal leachables from stainless steel. AAPS PharmSciTech 12 (1), 411–421. 10.1208/s12249-011-9592-3 21360314 PMC3066377

