# Supplementary figures and images for: Bioreactor as the root cause of the “manganese effect” during Aspergillus niger citric acid fermentations
Source: Front Bioeng Biotechnol. 2022 Aug 4;10:935902. doi: 10.3389/fbioe.2022.935902 (PMC9386146; doi:10.3389/fbioe.2022.935902)

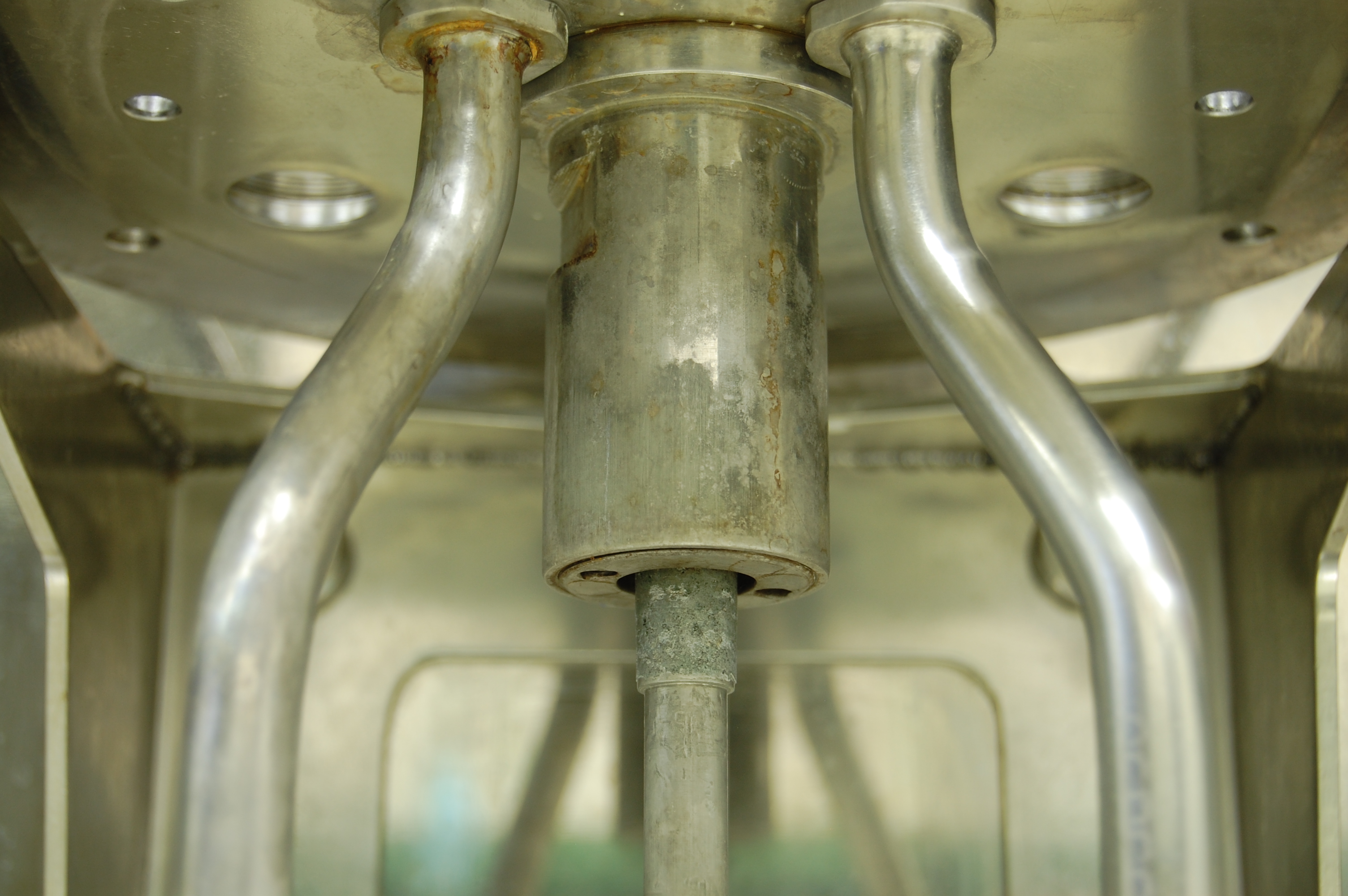

Supplement: Supplementary file 1 [file Image1.JPEG]
